# Inhibitory Effects of Arginine on the Aggregation of Bovine Insulin

**DOI:** 10.1155/2012/434289

**Published:** 2012-07-09

**Authors:** Michael M. Varughese, Jay Newman

**Affiliations:** The Department of Physics and Astronomy, Union College, Schenectady, NY 12308, USA

## Abstract

Static and dynamic light scattering were used to investigate the effects of L-arginine, commonly used to inhibit protein aggregation, on the initial aggregation kinetics of solutions of bovine insulin in 20% acetic acid and 0.1 M NaCl as a model system for amyloidosis. Measurements were made as a function of insulin concentration (0.5–2.0 mM), quench temperature (60–85°C), and arginine concentration (10–500 mM). Aggregation kinetics under all conditions had a lag phase, whose duration decreased with increasing temperature and with increasing insulin concentration but which increased by up to a factor of 8 with increasing added arginine. Further, the initial growth rate after the lag phase also slowed by up to a factor of about 20 in the presence of increasing concentrations of arginine. From the temperature dependence of the lag phase duration, we find that the nucleation activation energy doubles from 17 ± 5 to 36 ± 3 kcal/mol in the presence of 500 mM arginine.

## 1. Introduction

Conformational misfolding under destabilizing conditions and subsequent beta-sheet amyloid fibril formation has been shown to be a very common, perhaps generic, property of proteins [1, 2]. In a number of proteins, such aggregation is pathological leading to over 40 different neurodegenerative or systemic diseases [3, 4] such as Alzheimer's, Parkinson's, Huntington's, and type II diabetes. It is becoming more apparent that the toxic species of many proteins is formed in the early stages of soluble aggregation. Such proteins include A*β*, for which an annular protofibril form has recently been isolated [5], and insulin [6, 7]. Much recent effort has been devoted to understanding the details of aggregate formation and also to find ways to control or inhibit this process. In the case of insulin, the motivation for such studies includes improving its pharmacological use in treating diabetes, as well as using it as a model system for studying amyloid aggregation. 

Insulin, a key component in glucose metabolism, is a 51-residue protein consisting of two chains linked by disulfide bonds. Since the pioneering studies of David Waugh in the 1940's and 50's [8, 9], it has been known that, at elevated temperatures at low pH, insulin aggregates to form fibers that precipitate and/or form gels following a nucleation and elongation reaction. Despite this early finding and many subsequent studies [10–23], the molecular mechanism of insulin aggregation is not fully understood. 

Aggregation of insulin has been studied under a variety of solvent conditions and with added cosolutes. Added ethanol [24], metalloporphyrins [25, 26], targeted peptides [27], and proteins such as *α*-crystallin [28], *β*-casein [29], or heparin [30] have all been shown to have inhibitory effects on insulin aggregation. The addition of amino acids to insulin solutions also has been shown to inhibit aggregation and, of 15 amino acids studied, arginine was shown to be most effective [31]. Arginine has been studied as a suppressor of protein aggregation in a variety of protein systems [32–34]. The role of the guanidinium group of arginine binding to aromatic groups of insulin when these are exposed in misfolded monomers leading to inhibition of aggregation has been suggested by Lyutova et al. [35]. A recent paper by Shah et al. [36] reports that arginine can also act to promote aggregation of proteins and that the key as to whether it inhibits or promotes aggregation appears to be whether the guanidinium group binds to aromatic or acidic residues, respectively. 

In this work, we use dynamic and static light scattering to examine the effects of arginine on the early stages of thermally-induced insulin aggregation at low pH. We study the nucleation and early growth stages of insulin solutions in 20% acetic acid plus 0.1 M NaCl at varying concentrations of insulin and arginine and at various quench temperatures. From the temperature dependence of the nucleation lag time we are able to find the activation energy for nucleation of insulin in the absence and abundant presence (500 : 1 molar ratio) of arginine. We note explicitly that we have used light scattering rather than the more commonly used fluorescence of Thioflavin T to monitor aggregation since light scattering is more directly related to the weight average mass of the aggregates. 

## 2. Experimental Procedures

### 2.1. Materials

Solutions of bovine insulin (Sigma-Aldrich #15500, stored at −20°C) were prepared in 20% (v/v) acetic acid and 0.1 M NaCl using SuperQ water. Protein powder was dissolved at the appropriate concentration and spun at 5000 rpm in a JA-25.50 rotor of an Avanti Model J-25 centrifuge at 2°C for 5 minutes and then filtered, using a glass syringe and 13 mm diameter 0.22 micron pore-sized filters (Millex-GV) directly into precleaned 1 cm path length square optical glass cuvettes. These were stored at 2°C until used (within a few days). 

Filtered stock solutions of L-arginine (Sigma-Aldrich #A5006) were made in the same solvent as the insulin and stored at 2°C. Some insulin samples had appropriate volumetric additions of arginine to result in arginine concentrations ranging from 10 to 500 mM just prior to quenching at elevated temperatures in the light scattering thermal bath. 

### 2.2. Static and Dynamic Light Scattering

 Static and dynamic light scattering measurements were done using a home-made optical arrangement described previously [37], a Lexel argon ion laser operating in TEM_00_ mode at 514.5 nm and a Brookhaven BI-9000 correlator. A series of sequential two-minute experiments at a 90° scattering angle of both the average scattered intensity and the intensity autocorrelation function were made after insulin samples were placed in the thermal optical bath and quenched to the measurement temperature. Temperature equilibration occurred within two minutes at all quench temperatures. The intensity autocorrelation functions were analyzed by the method of cumulants to give an average hydrodynamic diameter of the scatterers in each experiment based on the Stokes-Einstein relation. Sequential experiments allowed us to follow the time dependence of the scattered intensity and the hydrodynamic diameter as the insulin aggregated. Control experiments with monodisperse polystyrene latex spheres assured that intensity autocorrelation function measurements correctly determined scatterer diameters. 

## 3. Results

**Figure 1:**
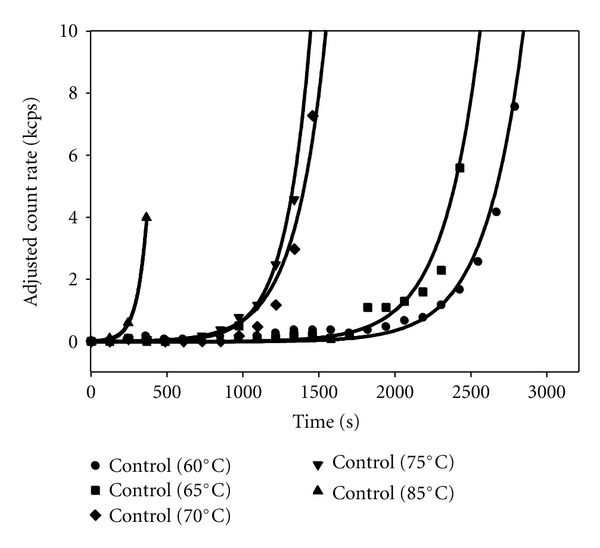
Average scattered intensity measurements versus time after quenching at varying temperatures for 1 mM insulin in 20% acetic acid + 0.1 M NaCl solutions. The count rates were measured in sequential two-minute dynamic light scattering experiments and, for purposes of comparison, the count rates were adjusted by subtracting the average initial count rates (typically several kcps). The smooth curves are fits to the data using (1) as described in the text. Similar data were obtained from plots of hydrodynamic diameter versus time after quenching to different temperatures, with the lag times typically somewhat shorter (10–30%) from these data.

**Figure 2:**
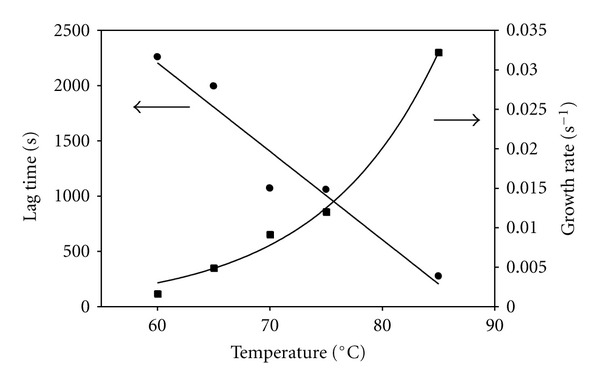
Data from Figure 1 were fit to two parameters, lag time and growth rate (using (1) as described in the text), and these are shown here as functions of the quench temperature. The smooth curves are linear and exponential fits to the data.

A series of light scattering measurements at a fixed insulin concentration (1 mM) were done at varying temperatures in the range of 60–85°C to study the thermal-induced aggregation of insulin. Note that the solvent was 20% acetic acid plus 0.1 M NaCl throughout this study and that it differed from the solvent in a recent previous study co-authored by one of us [12] by the addition of the salt. The presence of salt speeded up the kinetics dramatically and allowed us to study the inhibition effects of arginine on more reasonable time scales. At each constant temperature, static and dynamic light scattering measurements were made as a function of time (see Figure 1). The results from these measurements are characterized by a temperature-dependent lag time, followed by an exponential increase in scattered intensity and in hydrodynamic diameter (data not shown), obtained from simultaneous static and dynamic measurements, respectively. To obtain parameters describing these two phases of the aggregation, we fit data at each temperature to an exponential growth function of the form:

(1) I=exp⁡⁡[a(t−to)],

where *I* is the adjusted count rate and *t* is the time from quench, and the data were fit to two parameters: lag time, *t*_*o*_, and growth rate, *a*. The count rates were adjusted by subtracting the mean value of the initial count rate during the lag phase.  Figure 2 shows the dependence of these two parameters on quench temperature. At higher temperatures, the lag time becomes significantly shorter (by more than a factor of 8) with a linear dependence on quench temperature and the exponential growth rate becomes much faster (by close to a factor of 20). 

We point out that the data presented here are for the early stages in the aggregation process. Samples monitored for longer periods of time became inhomogeneous with large random fluctuations in intensity and hydrodynamic diameter, and upon removal of the cuvette from the thermal bath, it was apparent that a gel had formed. For the purposes of this study we only included the initial growth phase data and samples remained fairly homogeneous and in solution during all reported results. We performed all measurements two to four times, with resulting lag times and growth rates reproducing within about 10%. 

**Figure 3:**
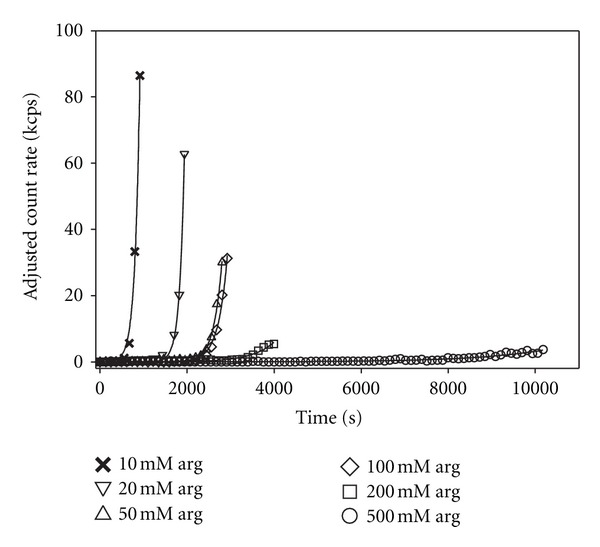
Adjusted (as in Figure 1 caption) average scattered intensity versus time after quenching to 70°C for 1 mM insulin with varying amounts of L-arginine added. Note the longer time scale (compared to Figure 1), the longer lag times, and the apparent slower growth rates at increasing arginine concentrations.

**Figure 4:**
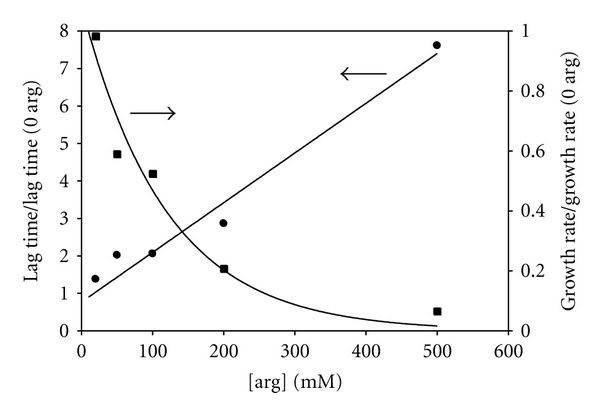
The lag time and growth rate, each normalized to the values with no added arginine, as functions of the amount of added arginine. The smooth curves are linear and exponential fits to the data.

To study the inhibitory effects of L-arginine on thermally-induced insulin aggregation, arginine was added to 1 mM insulin solutions in varying molar ratios from 10 : 1 to 500 : 1 and the time dependence of both the scattered intensity and hydrodynamic diameter were obtained at a fixed quench temperature.  Figure 3 shows the results of these measurements for the scattered intensity when samples were quenched to 70°C. Note the significantly lengthened time axis in this figure as compared to that in Figure 1, indicating that, in the presence of increasing amounts of arginine, the lag time increases. While less apparent from this figure, it can also be seen that the growth rate decreases with increasing arginine. This is made clearer in Figure 4 in which the lag time and the growth rate are shown, each normalized to the values in the absence of arginine, as functions of the arginine concentration. There is a linear growth in lag time and a nonlinear decrease in the growth rate with increasing arginine concentration. At the lowest arginine concentration used the lag time increases by 40% while the growth rate only slightly decreases by a few percent, while at 500 mM arginine the lag time increases by nearly a factor of 8 and the growth rate slows to only about 6.5% of its rate in the absence of arginine. 

A more limited set of results at both 0.5 mM and 2 mM insulin concentrations (not shown) show similar behavior with lag times that increase linearly with increasing arginine concentration. As a function of insulin concentration at a fixed arginine concentration, there is a monotonic decrease of approximately a factor of two in lag time with increasing insulin concentration from 0.5 to 2 mM. 

In the presence of arginine, we observed two distinct behaviors at later times (times beyond those reported in Figure 3) in the growth phase of the insulin aggregation. At arginine concentrations below 100 mM, we observed, similar to those samples in the absence of arginine, inhomogeneous scattering at these later times and a final gel-like appearance when examined by eye after removal from the temperature bath. For arginine concentrations of 100 mM and above, after the initial growth phase reported in Figure 3, precipitates formed and both sedimented to the bottom of the cuvette and collected at the meniscus due to surface tension. Also these samples had not formed a gel when examined after removal from the thermal bath. We reiterate that these late-stage behaviors are not presented further in this study.

**Figure 5:**
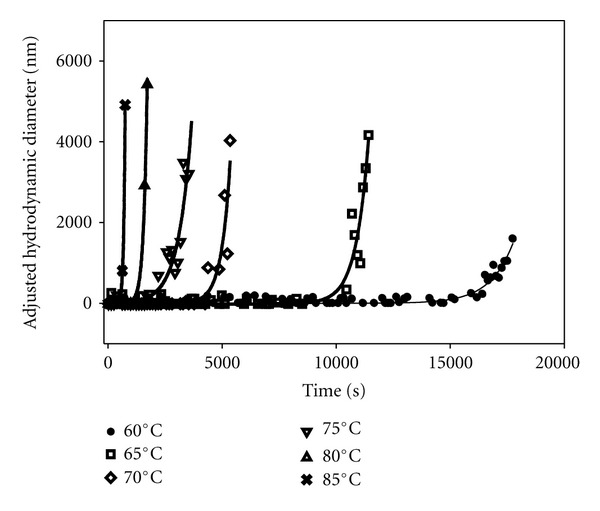
Hydrodynamic diameters of scattering species versus time for solutions of 1 mM insulin with 500 mM arginine at varying quench temperatures. The data are adjusted by subtracting the mean diameter during the lag time period in order to fit to the same two-parameter expression in (1). The smooth curves are fits to the same functional dependence as in (1) replacing intensity with hydrodynamic diameter.

**Figure 6:**
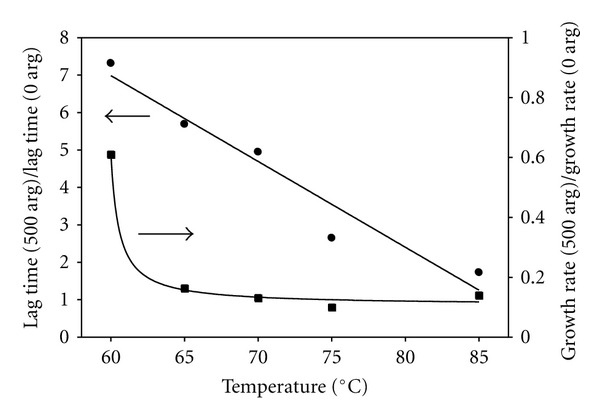
Lag times and growth rates of 1 mM insulin in the presence of 500 mM arginine, each normalized by the corresponding value in the absence of arginine, as function of the quench temperature. The smooth curves are linear and exponential fits to the data.

The temperature dependence of the inhibitory effect of L-arginine on insulin (1 mM) aggregation was studied at the fixed molar ratio of arginine to insulin of 500 : 1. As shown in Figure 5, as monitored by hydrodynamic diameter, at progressively lower quench temperatures the lag time increases and the growth rate decreases (as evidenced by the decreasing rate of slope change after the lag time). Again, we can quantitate this process by fitting the time dependence data to (1), with the intensity replaced by the hydrodynamic diameter, and extracting lag times and growth rates, and, in Figure 6, we show the normalized lag time and growth rate as functions of quench temperature. At this arginine concentration (500 mM), we see that the ratio of lag time with arginine to that without arginine decreases linearly with increasing temperature. At 60°C the lag time is roughly 7 times longer than that in the absence of arginine, while at 85°C the lag time is only about twice as long as that in the absence of arginine. Linear extrapolation to 37°C would predict a lag time that is over 12 times longer than in the absence of arginine at that temperature; these measurements were not attempted because of the extremely slow kinetics.

 The growth rates in the presence of 500 mM arginine increase monotonically with increasing temperature, as would be expected, by about a factor of 5 over our range of quench temperatures. On the other hand, the growth rate in the absence of arginine (see Figure 2) increases by about a factor of 20 over the same temperature range. Therefore, when plotted as the normalized growth rate (the ratio of growth rate with arginine to that in its absence) as in Figure 6, the normalized growth rate decreases nonlinearly from its value at 60°C and remains fairly flat at higher temperatures. In the presence of 500 mM arginine, the growth rate is roughly ten times slower than without arginine for most temperatures.

**Figure 7:**
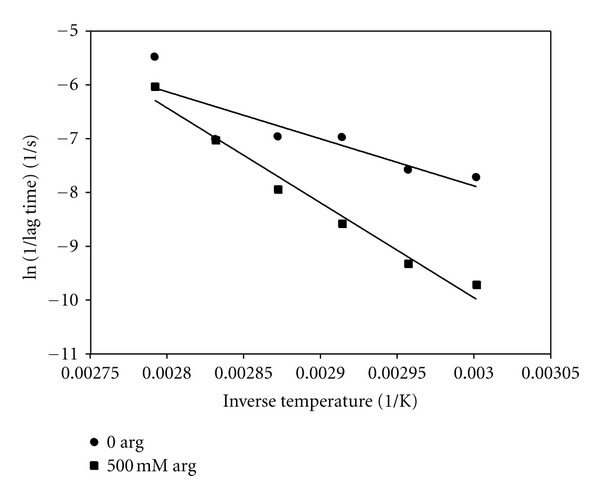
Arrhenius plots of the natural logarithm of the initial phase rate (taken as the inverse of the corresponding lag time) versus inverse absolute temperature for 1 mM insulin in the presence of and absence of 500 mM arginine.

From our data for the lag times at various quench temperatures in the absence and presence of arginine, we can make an Arrhenius plot using the inverse lag times as indicative of the nucleation rate:

(2) ln⁡⁡(1τ)=ln⁡⁡A−(ΔGR)(1T),

where *τ* is the lag time, Δ*G* is the nucleation free energy, *R* is the molar gas constant, and *T* is the absolute temperature, to determine Δ*G*. In Figure 7 we show this plot both for insulin alone and for insulin with 500 mM arginine. The negative slope of this plot is equal to the nucleation activation energy divided by the molar gas constant. We obtain an activation free energy in the absence of arginine of 17 ± 5 kcal/mol, and an activation free energy in the presence of 500 mM arginine of 36 ± 3 kcal/mol, an increase of more than a factor of two. 

## 4. Discussion

Under the proper destabilizing conditions, essentially all proteins are now believed capable of forming amyloid fibrils through a similar mechanism of aggregation leading to cross-*β* structure formation. Through this process, proteins may not only lose their biological functioning but may also become toxic leading to a variety of diseases. Insulin has long been known to form amyloid fibrils and has been extensively studied because of problems in its production, delivery, and storage for use in treating type II diabetes and also as a model system. 

The aggregation of insulin follows a nucleation-elongation process in which there is a lag phase during which monomers undergo a conformational change exposing hydrophobic residues and forming nuclei. It is known that in 20% acetic acid at ambient temperatures insulin is preferentially in the monomeric form, most susceptible to subsequent amyloidosis upon quenching to elevated temperature [16]. Recently the kinetics of amyloid growth has been modeled in terms of secondary, as well as primary, nucleation in which fragmentation and other secondary processes contribute to nuclei formation and to the kinetics [38]. This study found a strong correlation between the inverse lag time and growth rate based on their model. As we saw in Figure 2, for our insulin data we found this strong correlation: at increasing quench temperatures, lag time decreases and growth rate increases. After the lag phase, fibril growth has been shown to be exponential followed by gel-like formation and/or floccules formation, depending upon the conditions [11, 13]. 

In this study we have primarily studied the inhibitory effects of arginine on insulin aggregation. We chose, in these experiments, to add 0.1 M NaCl to the 20% acetic acid solvent in order to accelerate the aggregation kinetics, but not to agitate the samples (also known to accelerate the aggregation kinetics). Static and dynamic light scattering experiments were performed, in which the insulin concentration was varied between 0.5 and 2 mM, the arginine concentration varied from 10–500 mM, and the quench temperature ranged from 60 to 85°C. 

Control experiments in the absence of arginine (see Figures 1 and 2 for 1 mM insulin) show that the lag time decreases with increasing quench temperature and that the elongation phase has exponential growth with time, in qualitative agreement with previous studies which used a different solvent [12]. We also found that the elongation growth rate itself increases exponentially with quench temperature as shown in Figure 2. This same behavior was observed in a more limited data set at both 0.5 mM and 2 mM insulin with the lag time decreasing with increasing insulin concentration, in agreement with previous work [13]. Again we point out that the data reported here are only for the early elongation stage. Samples left longer at the quench temperatures showed large fluctuations in count rate and apparent hydrodynamic diameter and, when examined after removal from the thermal bath, had formed clear gels that did not flow at the higher insulin concentrations. 

Our data (see Figures 3 and 4) show that arginine acts as an effective inhibitor of insulin aggregation with lag times increasing linearly with increasing arginine concentration. The lag time increases by nearly a factor of 8 and the growth rate slows by about a factor of 15 at the highest arginine concentration compared to kinetics in the absence of arginine. We note that the average scattered intensity versus time and the apparent hydrodynamic diameter versus time (not shown) results are quite similar and give independent confirmation of the role of arginine in inhibiting insulin aggregation. 

The time course kinetics of both the average scattered intensity (not shown) and effective hydrodynamic diameter of insulin samples in the presence of 500 mM arginine (see Figures 5 and 6) show a characteristic temperature dependence. The normalized lag times (always longer than in the absence of arginine) decrease linearly with increasing temperature, with the ratio of lag time with arginine to that without arginine decreasing from a factor of nearly 8 at 60°C to only 1.7 at 85°C. These various experiments at different quench temperatures were further analyzed using an Arrhenius plot to obtain values for the nucleation free energy in the absence and presence of 500 mM arginine. We found about a two-fold increase in the activation energy for nucleation in the presence versus absence of arginine, with values of 36 and 17 kcal/mol, respectively. In the similar amyloid aggregating system of *A*β** there are two studies of the elongation kinetics that find values for the free energy associated with the fibril elongation process of 7 kcal/mol [39] and of 11 kcal/mol [40]. Assuming similar thermodynamics for the cross-*β* formation during aggregation, our values are somewhat larger than those, as might be expected, for the rate-limiting step of nucleation vs that of elongation. The significant increase in nucleation free energy in the presence of arginine emphasizes the inhibitory effect of arginine.

 Our results show the inhibitory effects of arginine on the initial aggregation kinetics of bovine insulin over a range of insulin concentrations and quench temperatures. While the mechanism of arginine's interaction with insulin and with other proteins is not yet well understood [32–36, 41, 42] it appears to be related to its binding to aromatic groups in partially unfolded states of proteins and may therefore be an effective inhibitory agent for a wide variety of amyloid forming proteins.

## References

[1] Dobson CM (2003). Protein folding and misfolding. *Nature*.

[2] Murphy RM, Kendrick BS (2007). Protein misfolding and aggregation. *Biotechnology Progress*.

[3] Chiti F, Dobson CM (2006). Protein misfolding, functional amyloid, and human disease. *Annual Review of Biochemistry*.

[4] Glabe CG (2008). Structural classification of toxic amyloid oligomers. *The Journal of Biological Chemistry*.

[5] Lasagna-Reeves CA, Glabe CG, Kayed R (2011). Amyloid-*β* annular protofibrils evade fibrillar fate in Alzheimer disease brain. *The Journal of Biological Chemistry*.

[6] Zako T, Sakono M, Hashimoto N, Ihara M, Maeda M (2009). Bovine insulin filaments induced by reducing disulfide bonds show a different morphology, secondary structure, and cell toxicity from intact insulin amyloid fibrils. *Biophysical Journal*.

[7] Heldt CL, Kurouski D, Sorci M, Grafeld E, Lednev IK, Belfort G (2011). Isolating toxic insulin amyloid reactive species that lack B-sheets and have wide pH stability. *Biophysical Journal*.

[8] Waugh DF (1946). A fibrous modification of insulin. I. The heat precipitate of insulin. *Journal of the American Chemical Society*.

[9] Waugh DF, Wilhelmson DF, Commerford SL, Sackler ML (1953). Studies of the nucleation and growth reactions of selected types of insulin fibrils. *Journal of the American Chemical Society*.

[10] Manno M, Craparo EF, Martorana V, Bulone D, San Biagio PL (2006). Kinetics of insulin aggregation: disentanglement of amyloid fibrillation from large-size cluster formation. *Biophysical Journal*.

[11] Mauro M, Craparo EF, Podestà A (2007). Kinetics of different processes in human insulin amyloid formation. *Journal of Molecular Biology*.

[12] Manno M, Giacomazza D, Newman J, Martorana V, San Biagio PL (2010). Amyloid gels: precocious appearance of elastic properties during the formation of an insulin fibrillar network. *Langmuir*.

[13] Nielsen L, Khurana R, Coats A (2001). Effect of environmental factors on the kinetics of insulin fibril formation: elucidation of the molecular mechanism. *Biochemistry*.

[14] Jiménez JL, Nettleton EJ, Bouchard M, Robinson CV, Dobson CM, Saibil HR (2002). The protofilament structure of insulin amyloid fibrils. *Proceedings of the National Academy of Sciences of the United States of America*.

[15] Hua Q, Weiss MA (2004). Mechanism of insulin fibrillation: the structure of insulin under amyloidogenic conditions resembles a protein-folding intermediate. *The Journal of Biological Chemistry*.

[16] Ahmad A, Uversky VN, Hong D, Fink AL (2005). Early events in the fibrillation of monomeric insulin. *The Journal of Biological Chemistry*.

[17] Krebs MRH, MacPhee CE, Miller A, Dunlop IE, Dobson CM, Donald AM (2004). The formation of spherulites by amyloid fibrils of bovine insulin. *Proceedings of the National Academy of Sciences of the United States of America*.

[18] Krebs MRH, Bromley EHC, Rogers SS, Donald AM (2005). The mechanism of amyloid spherulite formation by bovine insulin. *Biophysical Journal*.

[19] Librizzi F, Rischel C (2005). The kinetic behavior of insulin fibrillation is determined by heterogeneous nucleation pathways. *Protein Science*.

[20] Podestà A, Tiana G, Milani P, Manno M (2006). Early events in insulin fibrillization studied by time-lapse atomic force microscopy. *Biophysical Journal*.

[21] Grudzielanek S, Velkova A, Shukla A (2007). Cytotoxicity of insulin within its self-assembly and amyloidogenic pathways. *Journal of Molecular Biology*.

[22] Smith MI, Sharp JS, Roberts CJ (2008). Insulin fibril nucleation: the role of prefibrillar aggregates. *Biophysical Journal*.

[23] Pease LF, Sorci M, Guha S (2010). Probing the nucleus model for oligomer formation during insulin amyloid fibrillogenesis. *Biophysical Journal*.

[24] Dzwolak W, Grudzielanek S, Smirnovas V (2005). Ethanol-perturbed amyloidogenic self-assembly of insulin: looking for origins of amyloid strains. *Biochemistry*.

[25] Pasternack RF, Gibbs EJ, Sibley S (2006). Formation kinetics of insulin-based amyloid gels and the effect of added metalloporphyrins. *Biophysical Journal*.

[26] Sibley SP, Sosinsky K, Gulian LE, Gibbs EJ, Pasternack RF (2008). Probing the mechanism of insulin aggregation with added metalloporphyrins. *Biochemistry*.

[27] Gibson TJ, Murphy RM (2006). Inhibition of insulin fibrillogenesis with targeted peptides. *Protein Science*.

[28] Rasmussen T, Kasimova MR, Jiskoot W, van de Weert M (2009). The chaperone-like protein *α*-crystallin dissociates insulin dimers and hexamers. *Biochemistry*.

[29] Zhang X, Fu X, Zhang H, Liu C, Jiao W, Chang Z (2005). Chaperone-like activity of *β*-casein. *International Journal of Biochemistry & Cell Biology*.

[30] Giger K, Vanam RP, Seyrek E, Dubin PL (2008). Suppression of insulin aggregation by heparin. *Biomacromolecules*.

[31] Shiraki K, Kudou M, Fujiwara S, Imanaka T, Takagi M (2002). Biophysical effect of amino acids on the prevention of protein aggregation. *Journal of Biochemistry*.

[32] Ghosh R, Sharma S, Chattopadhyay K (2009). Effect of arginine on protein aggregation studied by fluorescence correlation spectroscopy and other biophysical methods. *Biochemistry*.

[33] Baynes BM, Wang DIC, Trout BL (2005). Role of arginine in the stabilization of proteins against aggregation. *Biochemistry*.

[34] Chen J, Liu Y, Wang Y, Ding H, Su Z (2008). Different effects of L-arginine on protein refolding: suppressing aggregates of hydrophobic interaction, not covalent binding. *Biotechnology Progress*.

[35] Lyutova EM, Kasakov AS, Gurvits BY (2007). Effects of arginine on kinetics of protein aggregation studied by dynamic laser light scattering and tubidimetry techniques. *Biotechnology Progress*.

[36] Shah D, Shaikh AR, Peng X, Rajagopalan R (2011). Effects of arginine on heat-induced aggregation of concentrated protein solutions. *Biotechnology Progress*.

[37] Newman J, Day LA, Dalack GW, Eden D (1982). Hydrodynamic determination of molecular weight, dimensions, and structural parameters of Pf3 virus. *Biochemistry*.

[38] Knowles TP, Waudby CA, Devlin GL (2009). An analytical solution to the kinetics of breakable filament assembly. *Science*.

[39] Kusumoto Y, Lomakin A, Teplow DB, Benedek GB (1998). Temperature dependence of amyloid *β*-protein fibrillization. *Proceedings of the National Academy of Sciences of the United States of America*.

[40] Carrotta R, Manno M, Bulone D, Martorana V, San Biagio PL (2005). Protofibril formation of amyloid *β*-protein at low pH via a non-cooperative elongation mechanism. *The Journal of Biological Chemistry*.

[41] Tsumoto K, Umetsu M, Kumagai I, Ejima D, Philo JS, Arakawa T (2004). Role of arginine in protein refolding, solubilization, and purification. *Biotechnology Progress*.

[42] Das U, Hariprasad G, Ethayathulla AS (2007). Inhibition of protein aggregation: supramolecular assemblies of Arginine hold the key. *PLoS ONE*.

